# Design of Peptidomimetic Functionalized Cholesterol Based Lipid Nanoparticles for Efficient Delivery of Therapeutic Nucleic Acids

**DOI:** 10.3390/molecules24183413

**Published:** 2019-09-19

**Authors:** Ehexige Ehexige, Tsogzolmaa Ganbold, Xiang Yu, Shuqin Han, Huricha Baigude

**Affiliations:** Institute of Mongolian Medicinal Chemistry, School of Chemistry & Chemical Engineering, Inner Mongolia University, Hohhot, Inner Mongolia 010020, China; ehexige@mail.imu.edu.cn (E.E.); Tsogi001@mail.imu.edu.cn (T.G.); xiangyu@mail.imu.edu.cn (X.Y.); chem-hshq@imu.edu.cn (S.H.)

**Keywords:** siRNA, lipid nanoparticle, peptidomimetic, cholesterol, Plk1

## Abstract

Lipid nanoparticles (LNP) are the most potent carriers for the delivery of nucleic acid-based therapeutics. The first FDA approved a short interfering RNA (siRNA) drug that uses a cationic LNP system for the delivery of siRNA against human transthyretin (hTTR). However, preparation of such LNP involves tedious multi-step synthesis with relatively low yields. In the present study, we synthesized cationic peptidomimetic functionalized cholesterol (denote Chorn) in straightforward chemical approaches with high yield. When formulated with helper lipids, Chorn LNPs complexed with siRNA to form nanoparticles with an average diameter of 150 nm to 200 nm. Chorn LNP mediated transfection of a green fluorescence protein (GFP) expressing plasmid resulted in 60% GFP positive cells. Moreover, Chorn LNP delivered siRNA against polo-like kinase 1 (Plk1), a disease related gene in cancer cells and efficiently suppressed the expression of the gene, resulting in significant morphological changes in the cell nuclei. Our data suggested that cholesterol based cationic LNP, prepared through a robust chemical strategy, may provide a promising siRNA delivery system.

## 1. Introduction

Since the discovery of RNA interference (RNAi) phenomena in *Caenorhabditis elegans* [1] and the subsequent proof of RNAi in mammalian cells by synthetic short interfering RNA (siRNA) [2], extensive studies have been carried out to potentiate the clinical application of siRNA drugs. The reason of such tremendous enthusiasm for development of siRNA drugs is that, unlike small molecular drugs, siRNA drugs can be designed to target any genes at a post-transcriptional level, and the cellular RNA-induced silencing complex (RISC) is so efficient that the IC50 value of siRNA is 100-fold lower than the value of antisense oligonucleotides (ASOs) [3]. However, RNAs are vulnerable in the circulation system, highly negatively charged with a relatively large molecular weight, and can potentially induce innate immune responses by binding and activating to pattern recognition receptors (PRRs) on cell surfaces or cytoplasm [4]. Therefore, a lack of efficient, protective, and targeted delivery systems has been the major bottleneck for the development of siRNA therapies [5].

Amongst the several types of materials currently being explored for the nucleic acid carrier, cationic lipids nanoparticles (LNP) are the most efficient ones. Cationic lipid formulations were used for gene (plasmid DNA) delivery [6]. Moderate gene transfer efficiency was observed for cationic liposome-mediated delivery of the cystic fibrosis (CF) transmembrane conductance regulator (CFTR) gene [7]. The similar cationic lipid-based formulations were adapted for the studies of siRNA delivery. Chemically modified siRNA [8,9] complexes with lipid bilayers consisting of cationic and fusogenic lipids to form so-called “stable nucleic acid-lipid particle” (SNALP), which dramatically increases the in vivo stability of siRNA. SNALP has been used as the carrier system for many pre-clinical and clinical studies of siRNA therapeutics, including siRNAs against viruses. For example, SNALP carrying siRNA against HBV successfully delivered the siRNA to the liver of mice carrying replicating HBV and significantly reduced serum HBV DNA, demonstrating the advances of cationic lipid-based siRNA carriers [10]; LNP-mediated delivery of siRNA against Ebola virus (EBOV) was able to protect 100% of nonhuman primates against lethal virus challenge [11]. In fact, the first-ever FDA approved siRNA drug Onpattro (Patisiran), uses SNALP as a delivery system for the treatment of transthyretin-mediated amyloidosis. So far, SNALP [12] is the most potent formulation comprised of a cationic lipid (DLin-MC3-DMA), cholesterol, helper phospholipid (DSPE), and PEG2000-C-DMG [13,14], in which the content of cholesterol is 40% (molar ratio). In fact, cholesterol is the essential element of the lipid nanoparticles and a key contributor to the cellular uptake of liposome-based particles. In mouse, injection of a cholesterol conjugated siRNA against apolipoprotein B (apoB) induced efficient knockdown of apoB in liver and decreased about 80% of apoB-100 protein in the plasma compared to the non-treated group [15]. OH-Chol, a cationic cholesterol derivative with a hydroxyethyl group at the amino terminus conjugated through a carboxamide-type linker, efficiently delivered siRNA in vitro and in vivo [16].

3β-[*N*-(*N*′,*N*′-dimethylaminoethyl)-carbamoyl] cholesterol (DC-Chol) is one of the most studied cationic cholesterol derivatives explored for the application of gene and siRNA delivery [17,18]. When formulated with dioleoyl phosphatidylethanolamine (DOPE), the transfection efficiency of the cholesterol-based cationic lipids was greatly improved [19]. Previously, we synthesized cholesterol-conjugated cationic lipids for RNAi delivery using microwave-assisted quaternization (MAQ) [20]. Although the cationic cholesterols showed excellent potency for in vitro siRNA delivery, the multiple steps synthetic approach was tedious with relatively low yields. In the previous work, we also discovered that the configuration of hydrophilic peptidomimetic headgroup of cationic lipids enhances biocompatibility of the resulting lipid nanoparticles (LNP) [21,22]. Peptidomimetics and peptides are potential carriers for siRNA. Conjugation of cell-penetrating peptides such as TAT peptide [23] and MPG [24] to siRNA can greatly enhance the cellular uptake of the siRNA. A fusion protein containing peptide transduction domain-dsRNA binding domain efficiently delivered siRNA to hard-to-transfect cells without eliciting immune responses in PBMCs [25]. A similar peptide-based siRNA delivery strategy was also demonstrated in RNAi-based anti-HIV study in vitro [26].

In the present report, we prepared a novel lipid material by conjugating short peptidomimetic headgroups to cholesterol (denotes Chorn). The procedures for synthesis of Chorn were straightforward with high yields, and the subsequent preparation of Chorn-based LNP was simple. Our research provides not only the demonstration of a robust synthetic strategy for peptidomimetic-lipid conjugates but also the evidence of promising DNA as well as siRNA delivery activities of such a design. While various peptides have been reported for gene delivery, this is the first report on cholesterol-based nucleic acid carrier having a short peptidomimetic as cationic group provider.

## 2. Materials and Methods

### 2.1. Chemicals

Cholesteryl chloroformate was purchased from TCI Chemicals (Shanghai, China). Benzotriazol-1-yl-oxytripyrrolidinophosphonium hexafluorophosphate (PyBOP) was purchased from J&K Chemicals Ltd. (Shanghai, China). Boc-Orn[(Boc-Orn(Boc-Orn(Boc)))-OH was purchased from GL Biochem Ltd. (Shanghai, China). 1,2-dioleoyl-sn-glycero-3-phosphocholine (DOPC), *N*,*N*-Diisopropylethylamine (DIPEA) and *N*-(3-Dimethylaminopropyl)-*N*′-ethylcarbodiimide hydrochloride (EDC) were purchased from Aladdin Chemicals (Shanghai, China). PEG2000-C-DMA was purchased from Sigma-Aldrich (St. Louis, MO, USA).

### 2.2. Synthesis of Intermediate **1**

To a solution of ethylenediamine (66.7 mg, 1.1 mmol) in dichloromethane (DCM, 5 mL), cholesteryl chloroformate (100 mg, 0.22 mmol) in DCM (5 mL) was slowly added dropwise, and the stirring continued at room temperature for 48 h; then, the solvent was removed by evaporation, and the target compound was purified by recrystallization using methanol to give 93.9 mg intermediate 1. The yield was 90.5%. MS: Calc. 472.40; Found: 495.40 (M+Na)^+^.

### 2.3. Synthesis of Chorn3

Intermediate product 1 (100 mg, 0.21 mmol) and Boc-Orn[(Boc-Orn(Boc-Orn(Boc)))-OH (160.91 mg, 0.21 mmol) were dissolved in dry dimethyl formaldehyde (DMF). After the solution was cooled in an ice bath, PyBOP (133.41 mg, 0.26 mmol) and DIPEA (27 mg, 0.21 mmol) were added and the reaction proceeded at room temperature under nitrogen protection for 48 h. After the reaction was complete, DMF was removed under reduced pressure, and the reaction mixture was suspended in DCM, followed by washing with 5% sodium citrate, saturated sodium chloride and water, and dried over anhydrous Na2SO4. The crude product was further purified by silica gel chromatography using DCM and methanol (8:1) as eluent to give a Chorn3 (223 mg, 86.9%). To remove Boc group, Chorn3 was dissolved in a HCl solution in 1,4-dioxane. After vigorously stirring at room temperature for 30 min, the solvent was evaporated, and the syrup was dried under vacuum in a desiccator containing NaOH to give deprotected Chorn3. MS: Calc. 814.64; Found: 815.65 (M+H)^+^.

### 2.4. Formulation of Chorn Lipid Nanoparticle

Chorn3 was mixed with DOPC and PEG2000-C-DMA at a ratio of 3:1:0.1, 4:1:0.1, 5:1:0.1, and 6:1:0.1 (weight/weight) in a solution of DCM and methanol (6:1), respectively. Then the solvent was removed under reduced pressure and the residue solid was dried under vacuum. PBS (1 X, pH = 7.4) was added to the dry residue so that the final concentration of each formulation reached 4.0 mg/mL and the suspension was incubated at r.t. for 1 h, followed by brief sonication and filtration (0.22 μm syringe filter). The formulated Chorn lipids were kept at 4 °C for further tests.

### 2.5. Electrophoresis Mobility Shift Assay

The Chorn3/siRNA complexes formed at various nitrogen/phosphate (N/P) molar ratios were confirmed by gel mobility shift assay. Nitrogen (N) represents the free amino groups on the Chorn3 lipids, and phosphate (P) represents phosphate group on RNA backbone. The N/P ratio was calculated by taking into account the molar concentration of free amino groups of Chorn3 and the phosphate content per siRNA (Table 1). Custom synthesized siRNA powder (Takara Biotechnology Co., Ltd. Dalian, Liaoning, China) was dissolved in RNase free water at a final concentration of 50 μM. 0.5 μL of stock solution of siRNA (50 μM) was added to 4.5 μL RNase free water to make a 5.0 μL solution. To this, a volume of Chorn3 formulation was added. The volume (X) of each Chorn3 formulation used to complex with siRNA at N/P 1:1 was calculated according to the formula:

[Conc. of stock Chorn3 formulation (0.45 μg/μL) × X × Chorn3 content in the formulation (%)]/molecular weight of Chorn3 (961.0 g/mol)/number of free amines in Chorn3 (4) = micromole of phosphate in siRNA (0.5 μM × 0.5 μL × 10^−6^ × 40 = 0.001 μmol).

N/P ratios 2, 3, 4, 5, and 6 were prepared by changing the volume proportion of Chorn3. The mixtures were incubated at r.t. for 20 min. Then siRNA loading buffer was added and the samples were run on a 1.0% agarose gel for 15 min at 100 V.

### 2.6. Measurements of Particle Size of Chorn3 Nanoparticles

Chorn3 (4 mg/mL, 20 μL in 1 X PBS, pH = 7.4) and complexed with 5 μL siRNA (50 μM) and the complexes were incubated for 20 min at room temperature. Then, 1 mL 1 X PBS (pH = 7.4) was added to the complex, and the particle size and zeta potential were measured on a Zetasizer Nano S instrument (Malvern Instrument, Malvern, UK). The final concentration of the Chorn3 was 80 μg/mL and siRNA was 250 nM, respectively. Used dip cell for zeta potential, measurement angle was 90° and measurement position (mm) was 4.50. All the sample attenuators were ranged from 6 to 11, dispersant RI was 1.330, viscosity (cP) was 0.882, dispersant dielectric constant was 78.5, and temperature was 25.0 °C.

### 2.7. Transmission Electron Microscopy (TEM)

Shape and surface morphological examination of siRNA/Chorn3 nanoparticles was observed by transmission electron microscopy (TEM). A mixture of Chorn3 (5:1) and siRNA was incubated at room temperature for 20 min, two drops of sample were placed on a copper grid and air-dried for 10 min, and then negatively stained with 2% phosphomolybdic acid solution for 2 min. The grid was allowed to air-dry for 10 min and examined under the TEM (Instrument: FEI Tecnai G2 F20. Voltage: 200 kV).

### 2.8. Confocal Imaging

HeLa cells were cultured on glass coverslips to 50–60% confluence. The cells were transfected with Chorn3/polo-like kinase 1(Plk1)-siRNA complexes in serum-free medium for 4 h at 37 °C and 500 μL of DMEM medium containing 10% FBS and 1% penicillin/streptomycin was added. Final concentrations of Plk1-siRNA and Chorn3 nanoparticles were 100 nM and 32 μg/mL, respectively. After the cells were further incubated 20 h, the cells were washed 5–6 times with PBS (1 X, pH = 7.4) and fixed with 4% paraformaldehyde (PFA) for 20–30 min at RT. After rinsing the slides with PBS 3 times, the fixed cells were stained with DAPI for 10 min. The coverslips were detached and mounted on glass slide by mounting media and the image was captured by confocal laser-scanning microscopy (Fluoview FV 1000, Olympus Corporation, Tokyo, Japan).

### 2.9. Cell Culture and Cytotoxicity Assay

293T cells, HeLa cells, and HepG2 cells (ATCC, Manassas, VA, USA) were maintained at 37 °C with 5% CO2 in DMEM supplemented with 10% fetal bovine (HyClone), 100 U mL^−1^ penicillin and 100 μg mL^−1^ streptomycin. Cells were seeded in 96-well plates at 5,000 cells/well in 100 μL of culture medium. After 24 h of seeding, Chorn3 lipid nanoparticles (4 mg/mL) were added to the cells at final concentrations of 4, 8, 20, 60, and 100 μg/mL in triplicate. After 24 h, cell viability was assessed using MTT assay.

### 2.10. Transfection Efficiency

For cellular uptake assay, the fluorescence signal was measured by flow cytometry assay. Lipofectamine 2000 (L2K) was used as the comparison group. A total of 2.5 × 10^6^ Hela cells were treated with FITC-siRNA/Chorn3 in a 12-well plate for 6 h. The final concentration of Chorn3 and FITC-siRNA were 32 μg/mL and 50 nM, respectively. After that cells were washed three times with PBS (1 X, pH7.4) to remove excess FITC-siRNA, all samples were finally acquired on an ACEA NovoCyteTM Benchtop Flow Cytometer and analysis by NovoExpression software (ACEA Biosciences, Inc., San Diego, CA, USA).

For plasmid transfection, 1 μg pcDNA3-e green fluorescence protein (GFP) was diluted in 100 μL Opti-MEM. This solution was mixed with 100 μL Opti-MEM containing Chorn3 lipids, and incubated for 20 min before adding to the cells in 800 μL complete medium in a well of 12-well plate. The final concentration of Chorn3 in the well was 32 μg/mL. After 48 h, the GFP expression was evaluated by observation under fluorescence microscope. For flow cytometric analysis (FCM), cells were trypsinized, washed, and resuspended in PBS and assessed on a NovoCyte Benchtop Flow Cytometer (ACEA Biosciences, Inc., San Diego, CA, USA).

For transfection of siRNA, siGenome Non-targeting siRNA Control was purchased from Dharmacon (Lafayette, CO, USA). Plk1 siRNA was custom synthesized by Takara Biotechnology Co., Ltd. (Dalian, Liaoning, China). The sequence of Plk1 siRNA: sense strand: 5′-GCACAUACCGCCUGAGUCUUU-3′; antisense strand: 5′-AGACUCAGGCGGUAUGUGCUU-3′ [27]. Cells were seeded in a 12-well plate at 2.5 × 10^6^ cell/well density and cultured for 24 h; when cell confluency reached 65%, cells were transfected with either Chorn3/Plk1 siRNA complex, or Chorn3/siGenome non-targeting siRNA Control, at siRNA concentrations of 25 nM, 50 nM, and 100 nM, respectively. The final concentration of Chorn3 in the well was 32 μg/mL. After 24 h, total RNA was extracted using TRIZOL (Invitrogen, Carlsbad, CA, USA) and the expression of Plk1 mRNA was measured with iScriptTM Reverse Transcription Supermix for RT-qPCR and iTaqTM Universal SYBR Green Supermix (Bio-Rad, Hercules, CA, USA) for quantitative PCR. The sequence of primers used for RT-qPCR were as following: human Plk1, forward: 5′-cacagtgtcaatgcctcca-3′, reverse: 5′-ttgctgacccagaagatgg-3′, human β-actin, forward: 5′-ccaaccgcgagaagatga-3′, reverse: 5′-ccagaggcgtacagggatag-3′.

### 2.11. Statistical Analysis

The data were expressed as the means ± SEM and were analyzed for significant differences by one-way analysis of variance (ANOVA) with Dunnett’s multiple comparisons test using GraphPad Prism 7.0. Differences were considered statistically significant if *p* value is < 0.05.

## 3. Results

### 3.1. Synthesis and Characterization of Chorn3 Lipid

**Scheme 1:**
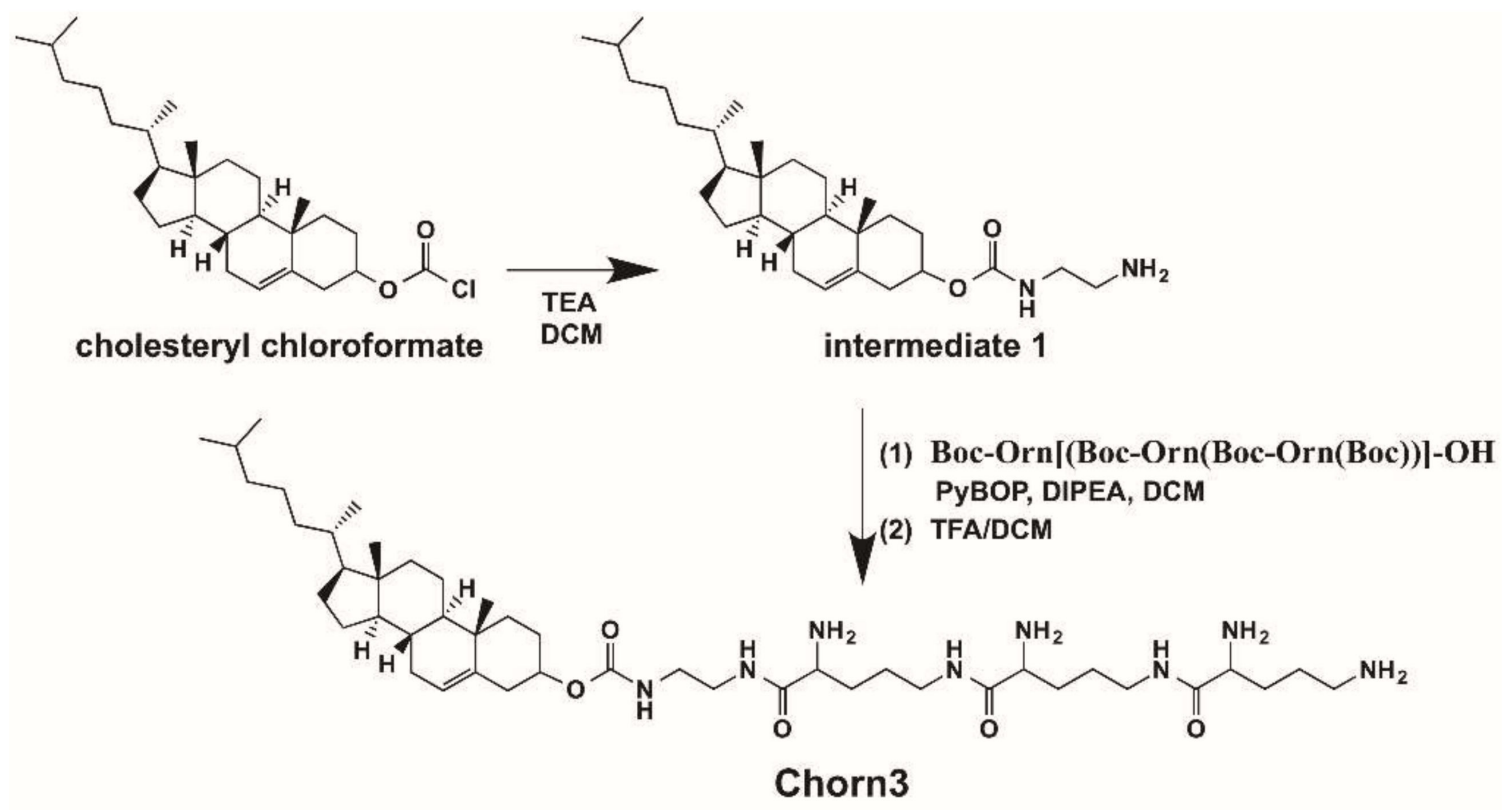
Schematic diagram of the synthesis of cationic peptidomimetic functionalized cholesterol (Chorn)3.

**Figure 1:**
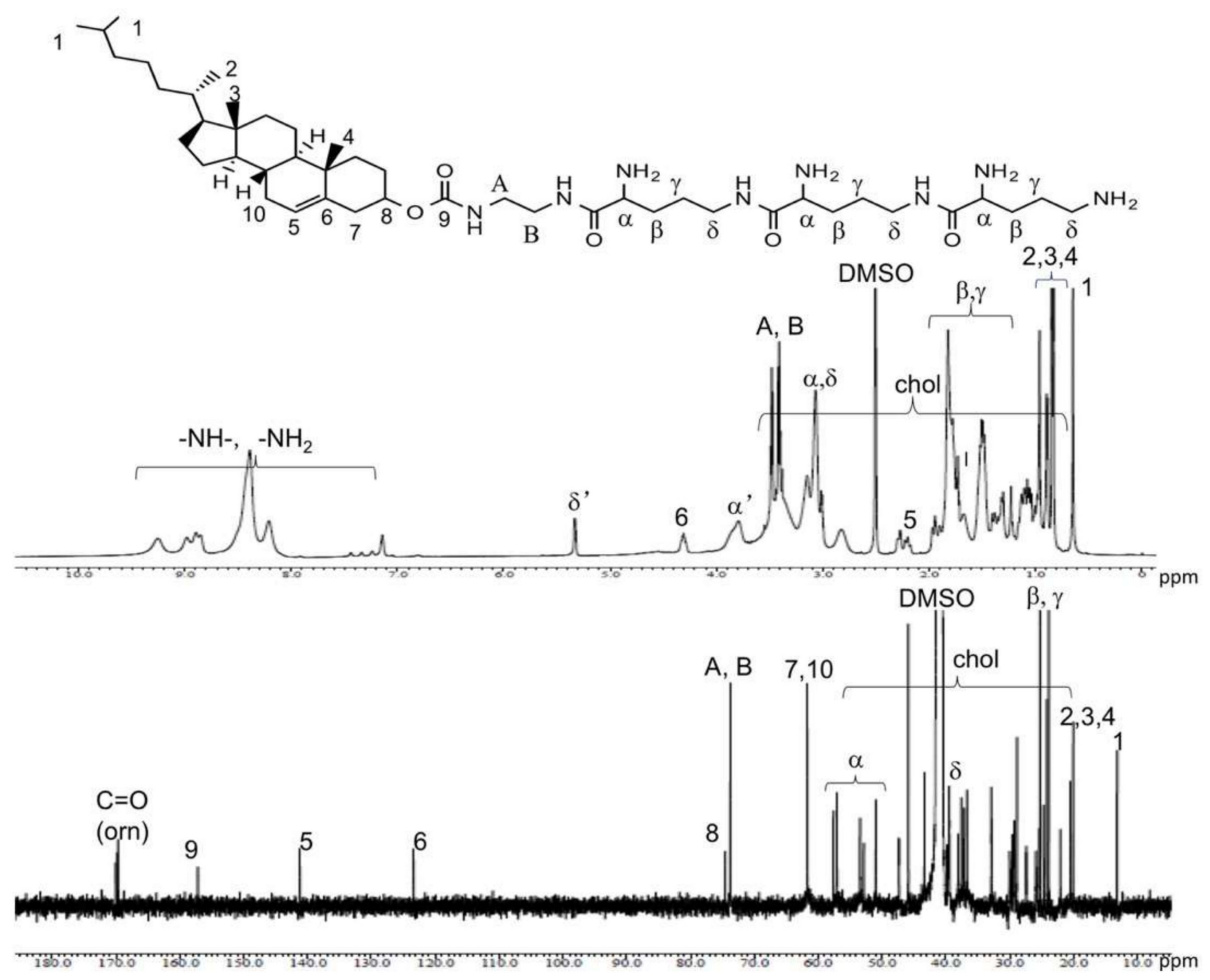
Characterization of Chorn3 lipid by ^1^H-NMR (**upper**) and ^13^C-NMR (**lower**) spectra in DMSO-*d*_6_.

We coupled ethylenediamine with a more reactive cholesteryl chloroformate to prepare an intermediate 1 with a free amino group (Supporting Information Figure S1), which readily reacted with the carboxyl group of the peptidomimetic when PyBOP was used as a condensing reagent to give Chorn3 (Scheme 1) in an appreciable yield. The Structural analysis by NMR confirmed that the designed Chorn3 was successfully synthesized (Figure 1).

### 3.2. Formulation and Physicochemical Characterization of Chorn3 LNP

**Figure 2:**
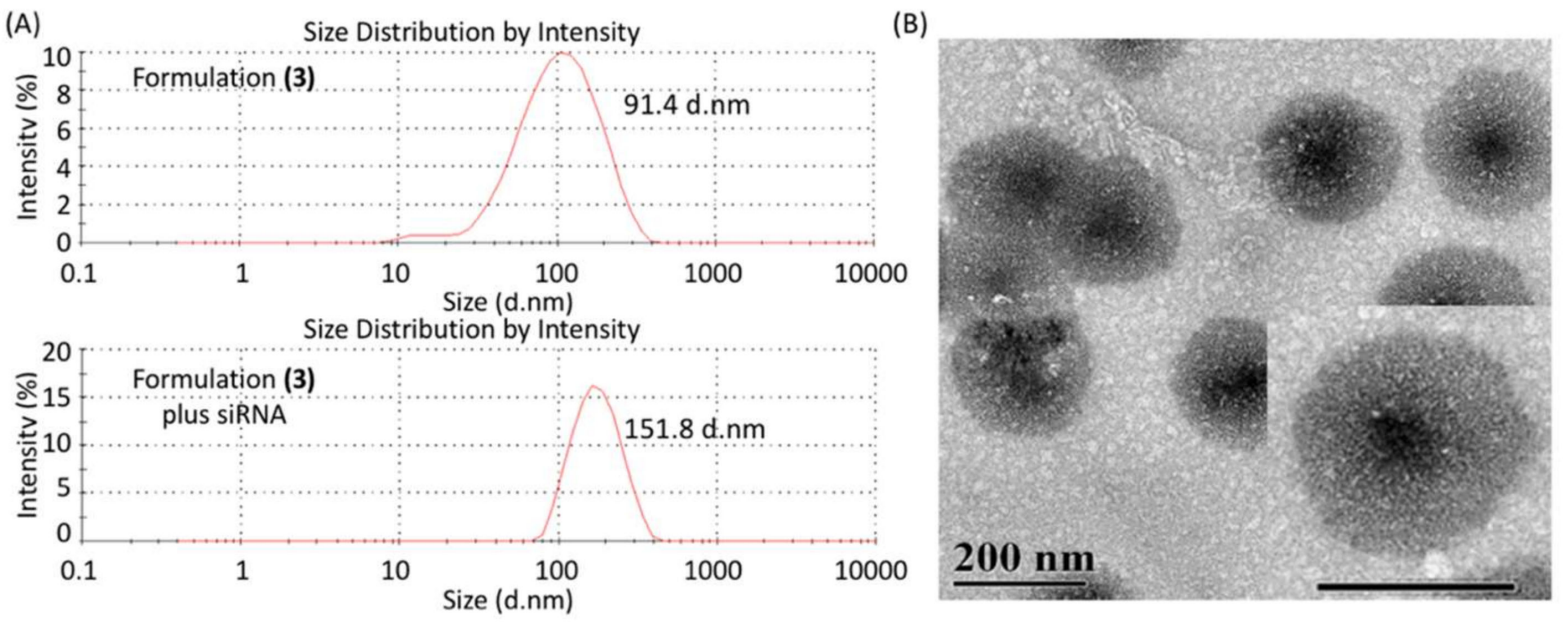
Measurement of the size distribution of Chorn3 LNP (3). (**A**) Size distribution obtained by dynamic light scattering (DLS) before and after complexing with short interfering RNA (siRNA). (**B**) TEM image of Chorn3 LNP (3) complexed to siRNA.

Next, we formulated Chorn3 by incorporating neutral helper lipid DOPC at different ratios. PEG2000-C-DMA was also included in all the formulations at a 10% ratio (weight). The mixture of the components was resuspended in PBS (1 X, pH7.4) at a final concentration of 4 mg/mL. All formulations were stable at room temperature after a brief sonication and subsequent filtration. The physicochemical characterization of the formulations for the particle size, polydispersity index (PDI) and zeta potential are summarized in Table 2. Binding of Chorn3 formulation to siRNA resulted in formation of lipid nanoparticles with diameter about 150 nm to 200 nm (Figure 2, Figure S2), with zeta potential of about 26 to 33 mV (Table 2, Figure S3), indicating that we have successfully prepared a cholesterol-based cationic LNP.

### 3.3. Electrophoresis Mobility Shift Assay

**Figure 3:**
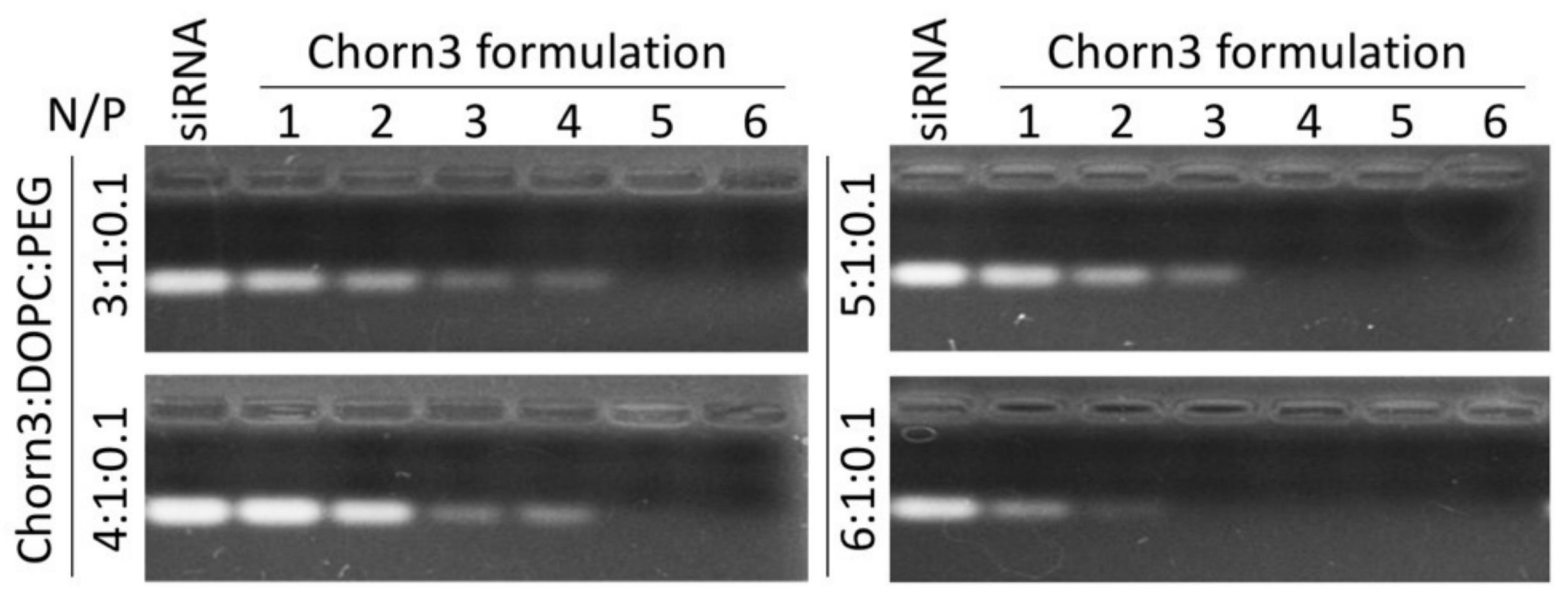
Chorn3 formulations bind to siRNA and form nanoparticles. Electrophoresis mobility shift assay for Chorn3 formulations complexed to siRNA. Lane 1, free siRNA. Lane 2–7, Chorn3 complexed siRNA at nitrogen: phosphate ratio (N/P) of 1, 2, 3, 4, 5, and 6, respectively.

To assess the RNA binding ability of the Chorn3 LNP, we mixed siRNA with different Chorn3 formulations, respectively, incubated for 20 min and analyzed the unbound residue siRNA on agarose gel (Figure 3). The siRNA binding ability of Chorn3 formulations increased with the increase of Chorn3 lipid content in the formulation, with the order of the binding strength as following: 6:1 > 5:1 > 4:1 = 3:1.

### 3.4. Cytotoxicity Assay

**Figure 4:**
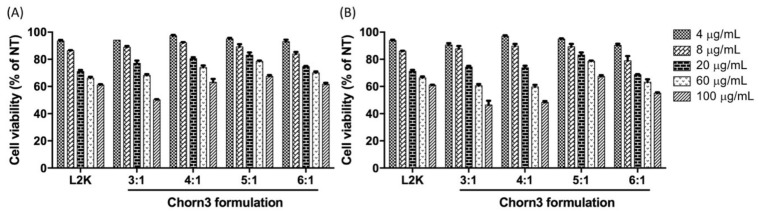
Cytotoxicity assay for Chorn3 formulation. (**A**), HeLa cells (**B**), HepG2 cells.

To measure the cytotoxicity of Chorn3 formulations, we treated three different cell lines, i.e., HEK293 cells, HepG2 cells, and HeLa cells, with increasing concentrations of the formulations, and measured the cell viability by MTT assay (Figure 4). All three formulations showed more cell viability than lipofectamine 2000 did, with formulation 5:1 being the least toxic to all three cell lines. At the highest concentration used for the MTT assay, treatment of the cells with formulation 5:1 at 100 μg/mL concentration resulted in more than 80% cell viability, suggesting that the novel cholesterol-based LNPs may be used for siRNA delivery.

### 3.5. Cellular Uptake Assay

**Figure 5:**
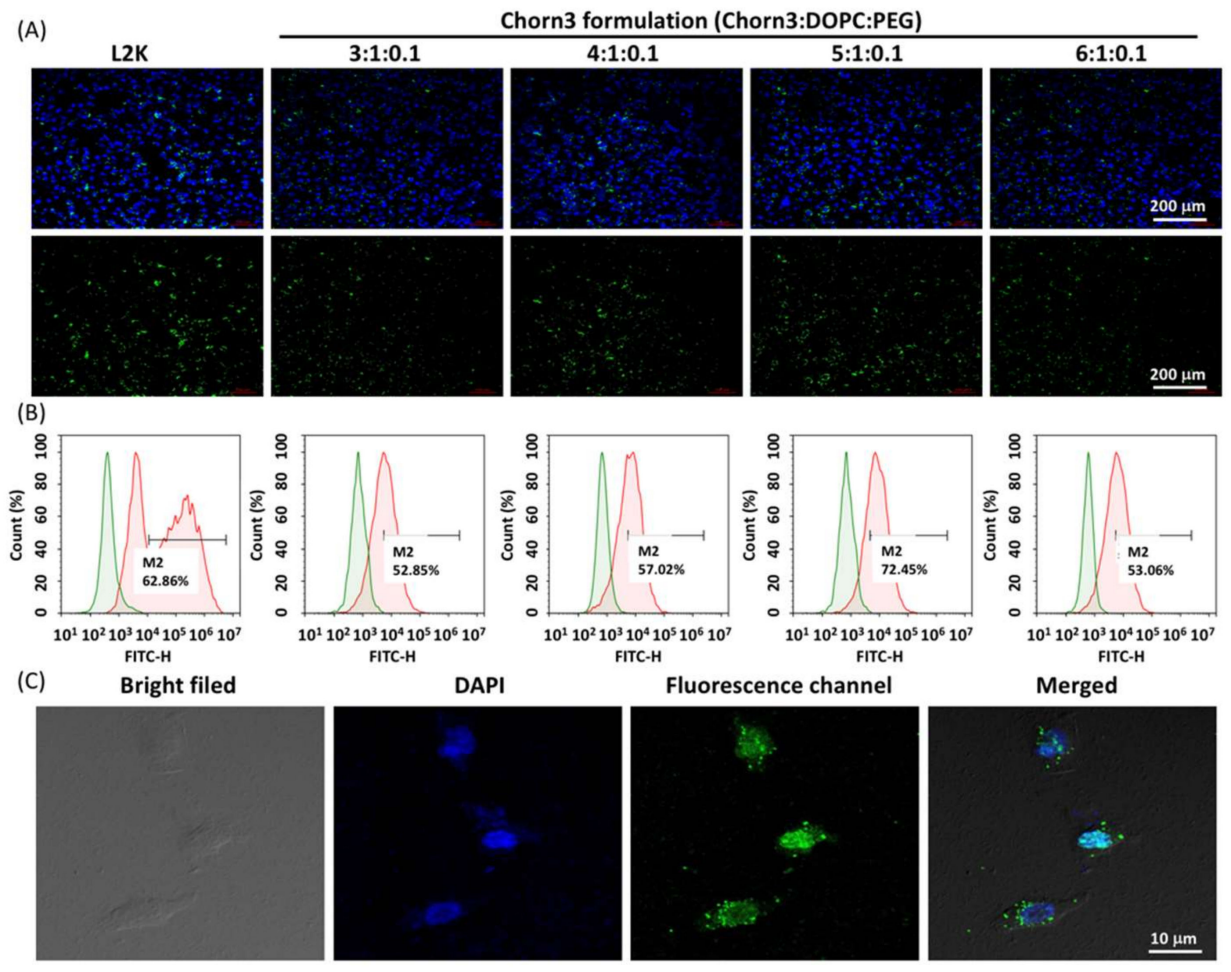
Measurement of cellular uptake of siRNA delivered by Chorn3 LNP. (**A**) Fluorescence microscopy observation of HeLa cells treated with FITC-labeled siRNA complexed to lipofectamine 2000, or different formulations of Chorn3 LNP; (**B**) quantification of FITC-positive cells by flow cytometry; (**C**) confocal images of HeLa cells transfected with FITC-labeled siRNA complexed to Chorn3 formulation (5:1:0.1). Image size: 100 × 100 μm.

Next, we assessed the cellular uptake of the siRNA delivered by Chorn3 LNP. To do this, we treated HeLa cells with FITC-labeled siRNA complexed to Chorn3 LNP. Then, we observed the fluorescence signal in the cells by fluorescence microscopy. The fluorescence signal was detected in all treatment groups. The strongest FITC signal was detected in the cells treated with FITC-siRNA/Chorn3 (5:1) (Figure 5A). Subsequent quantification of FITC-positive cells by flow cytometry revealed that 72.4% cell internalized the siRNA delivered by Chorn3 LNP formulation 5:1 (Figure 5B), suggesting that Chorn3 LNPs can efficiently deliver siRNA into the cells. Interestingly, confocal imaging of HeLa cells transfected with Chorn3 formulation (5:1:0.1) revealed that the majority of the FITC signal was detected in the nuclei (Figure 5C), indicating that Chorn3 LNP mediated nucleic acid delivery may target nuclei.

### 3.6. Plasmid Transfection

**Figure 6:**
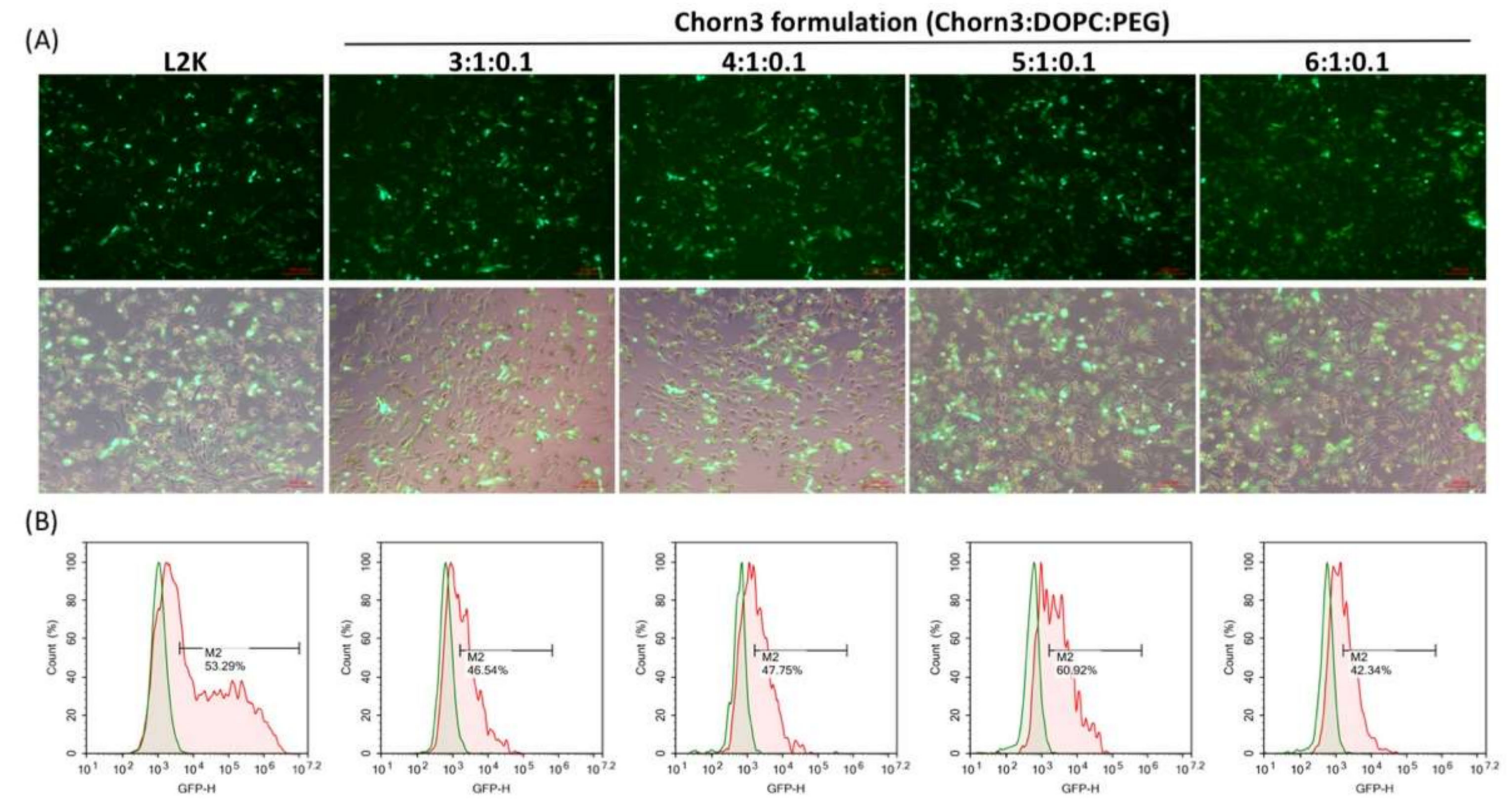
Plasmid transfection by Chorn3 LNP. (**A**) Fluorescence microscopy images of HEK293T cells transfected with pcDNA-eGFP plasmid complexed to lipofectamine 2000 (L2K) or Chorn3. (**B**) Quantification of GFP expressing cells by flow cytometry. HEK293T cells were transfected with pcDNA-eGFP plasmid complexed to Chorn3 formulations. 24 h later, GFP expressing cells were quantified by flow cytometry.

To evaluate the nucleic acid transfection efficiency of Chorn3 formulations, we complexed pcDNA3-eGFP plasmid with Chorn3 formulations, respectively, and treated HEK293 cells. After 48 h, the expression of GFP was observed by fluorescence microscopy (Figure 6A) and the GFP expressing cells were quantified by flow cytometry (Figure 6B). All four formulations showed plasmid delivery efficiency, of which Chorn3 formulation 5:1 had the highest efficiency. Compared to lipofectamine 2000 (53.29%), transfection by Chorn3 formulation 5:1 resulted in 60.92% GFP positive cells, indicating that, in this experimental setting, the DNA transfection efficiency of Chorn3 formulation 5:1 is better than that of lipofectamine.

### 3.7. siRNA Delivery Assay

**Figure 7:**
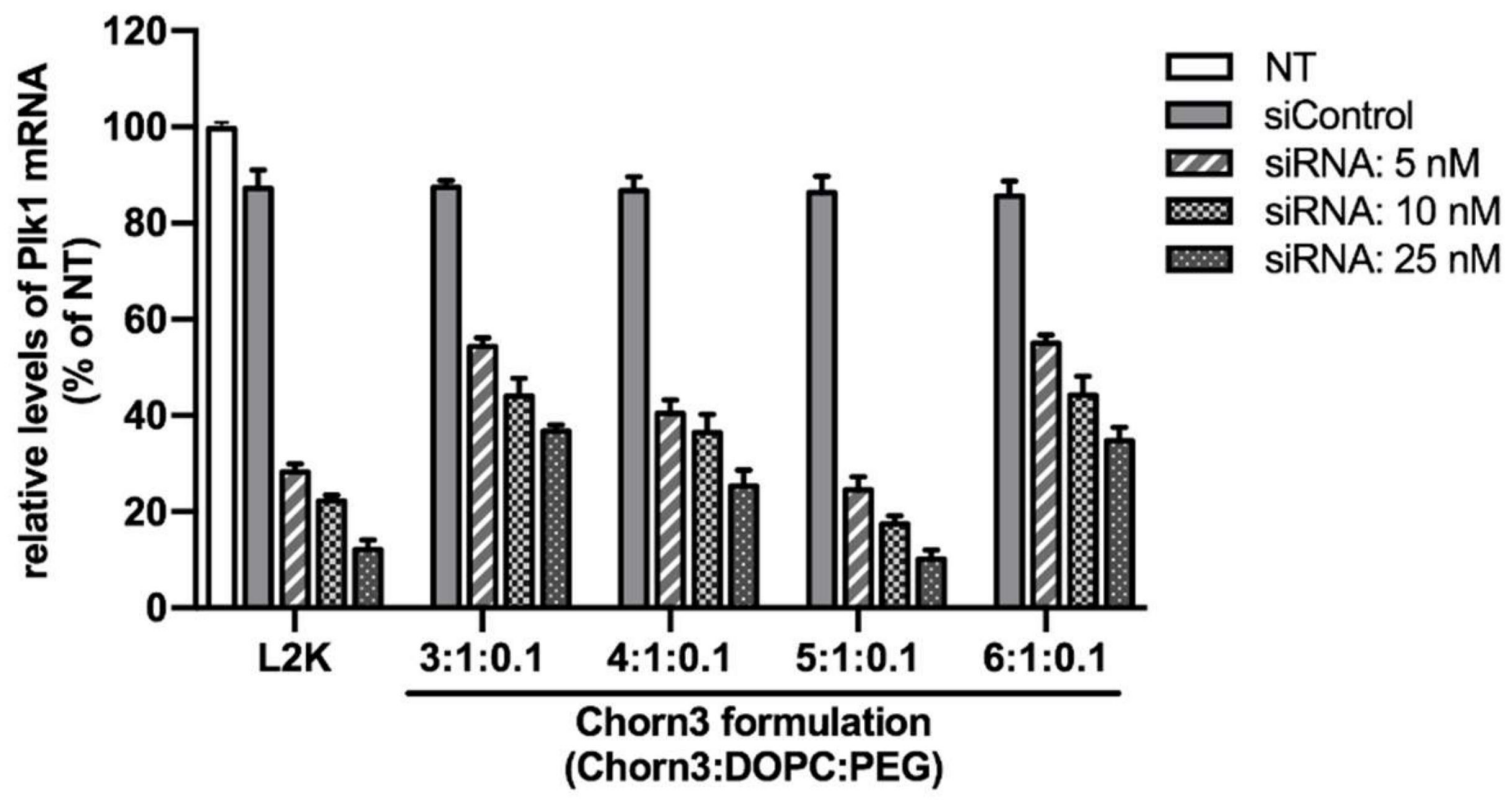
siRNA delivery efficiency of Chorn3 formulations. Error bars illustrate s.d. of the mean.

Next, we investigated the siRNA delivery efficiency of Chorn3 LNPs. To do this, we treated HeLa cells with different concentrations of siRNA against Polo-like kinase-1 (Plk1) complexed to Chorn3 LNPs or lipofectamine 2000, respectively, and analyzed the mRNA level of Plk1 in total RNA extracted from cells 24 h after the transfection. RT-qPCR analysis revealed that, the siRNA transfection efficiency of Chorn3 LNP increased with the increase of siRNA concentration, and at the highest siRNA concentration (final concentration of 25 nM) tested, the efficiency of Chorn3 LNP mediated knockdown of Plk1 was as follows: Chorn3 (3:1), 64%; Chorn3 (4:1), 75%; Chorn3 (5:1), 90%; Chorn3 (6:1), and 65% (Figure 7). Again, Chorn3 (5:1) LNP showed the highest siRNA transfection efficiency in all three siRNA concentrations tested, which is consistent with the plasmid transfection (Figure 6).

### 3.8. Phenotype of Plk1 Knockdown by Chorn3 LNP Mediated siRNA Delivery

**Figure 8:**
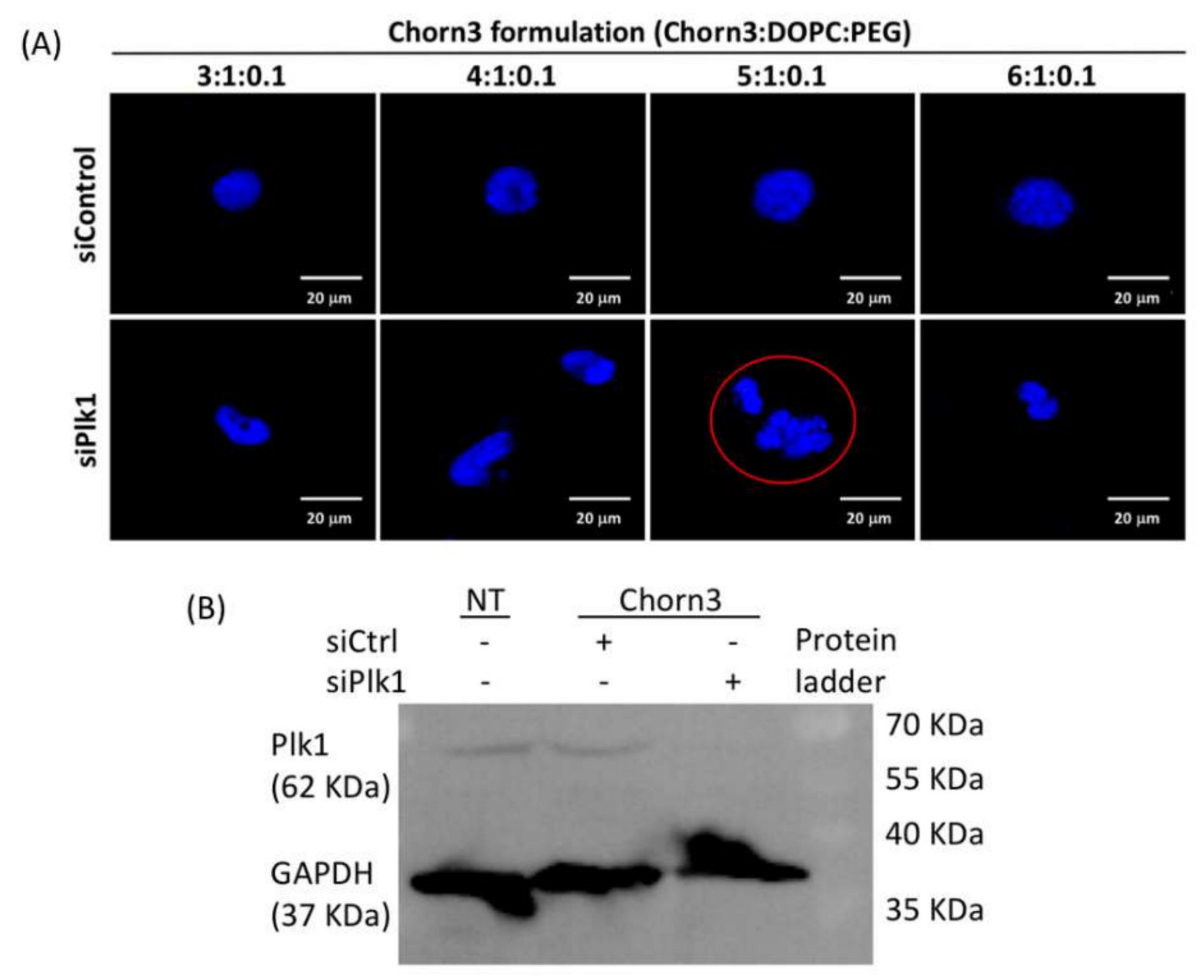
Chorn3 mediated delivery of siRNA against polo-like kinase 1 (Plk1) in HeLa cells. (**A**) Confocal images showing segregation of nuclei (red circle) induced by RNA interference (RNAi) mediated silencing of Plk1 gene. (**B**) Western blotting showing significant decrease of Plk1 protein in cells transfected with siPlk1/Chorn3 (5:1).

Plk1 regulates centrosome duplication and maturation. Inhibition of Plk1 induces abnormal centrosome amplification, which leads to multipolar spindles and unequal segregation of chromosomes. Therefore, Plk1 inhibition is considered to be a promising therapeutic strategy [28,29,30]. Treatment of HeLa cells with siPlk1/Chorn3 led to improper DNA condensation in the cell nucleus (Figure 8), suggesting an aborted mitotic arrest induced by silencing of Plk1 at the protein level. The unequal segregation of chromosome was most obvious in siPlk1/Chorn3 (5:1) treated cells, which is consistent with the quantification of Plk1 mRNA level by RT-qPCR (Figure 6). In contrast, the treatment with siControl/Chorn3 complex did not induce any obvious morphologically changes in the nuclei, indicating that the morphological changes were not induced by the carrier (Chorn3 LNP).

## 4. Discussion

Previously, we discovered that the type and combination of amine in the headgroup of cationic lipids have a great influence on not only the nucleic acid delivery ability but also the cytotoxicity of the resulting lipid nanoparticles [22]. A headgroup containing multiple ornithine residues linked through an amide bond between delta-amine and the alpha carboxyl group showed the most potency for the creation of lipid functionalized peptide or peptidomimetic based siRNA delivery system [21,22]. Our initial attempts for creation of peptidomimetic conjugated cationic lipids also included lysine and arginine residues in the headgroup. However, only the ornithine based peptidomimetics showed significantly higher transfection efficiency. To create easy-to-make lipid nanoparticles and explore their potential application for in vitro siRNA delivery, we conjugated ornithine peptidomimetics to cholesterol by straightforward ester or amide formation reaction. Our initial attempts to couple cholesterol directly to Boc-Orn[(Boc-Orn(Boc-Orn(Boc)))-OH through ester bond gave very low yield. Moreover, the nature of linkage between cholesterol and the cationic headgroup determines the gene transfection efficiency of the resulting lipids [31]. Therefore, we introduce a short linker to prepare an intermediate 1 containing a free amine, which easily formed amide bond with carboxyl group in the peptidomimetic. The resulting lipid Chorn3, after deprotection, gives three alpha-amine and a terminal delta-amine (Scheme 1).

Cationic lipids are often formulated with helper lipids to increase the stability in circulation, enhance the nucleic acid delivery efficiency and improve the endosomal release of the cargo from the lipid nanoparticles [32]. Therefore, we formulated the Chorn3 lipid by incorporating neutral lipid DOPC as well as PEG2000-C-DMA. The size distribution obtained by DLS and TEM revealed that Chorn3 Formulation (3) LNP has an average diameter of about 150 nm, which is the smallest of all four formulations, and a zeta potential of about 33 mV, the largest in all four formulations (Table 2, Figure 2).

One of the preconditions for encapsulation of nucleic acid by LNPs is the effective binding, which is based on the electrostatic interactions between negative charges of nucleic acid and the positive charges from cationic lipids. The affinity of Chorn3 LNP to siRNA was assessed by electrophoresis mobility shift assay. At an N/P ratio of 5, Formulations (1) and (2) completely inhibited mobility of the siRNA, whereas Formulations (3) and (4) required lesser N/P ratios of 4 and 3, respectively (Figure 3), indicating that the increased ratio of Chorn3 lipid in the formulation enhances the siRNA binding ability of the resulting LNP. Next, we measured the toxicity of all Chorn3 formulations on two cell lines (HeLa cells and HepG2 cells) by MTT assay. The cell viability after treatment with Formulations (1) and (2) at the range of concentrations used for the MTT assay was similar to that of lipofectamine 2000 (Figure 4). However, Formulation (3) showed significantly higher cell viability in both cell lines, especially at concentrations 60 μg/mL and below, which is in the range of in vitro transfection of plasmid and siRNA (the maximum concentration of Chorn3 LNP used for in vitro plasmid and siRNA transfection was 32 μg/mL).

Efficient cellular uptake and subsequent endosomal release of cargo are crucial to the successful intracellular delivery of nucleic acid therapeutics. To evaluate the cellular uptake of siRNA complexed to Chorn3 LNPs, we treated HeLa cells with a FITC-label siRNA sequence complexed to Chorn3 LNP formulations, respectively. The observation of the treated cells with fluorescence microscopy revealed that all four formulations effectively delivered siRNA into the cells, with Formulation (3) showing the highest FITC positive cell population, as measured by flow cytometry (Figure 5). Confocal imaging revealed that the FITC signal from Formulation (3) treated cells were located in both nucleus and cytoplasm 24 h post-transfection (Figure 5C). The transfection of GFP plasmid into HeLa cells by Chorn3 formulations also induced GFP expression. Again, Formulation (3) gave the highest GFP positive cells (Figure 6), indicating that Chorn3 can effectively protect the DNA from enzymatic hydrolysis in the cytoplasm and facilitates the nuclear localization of plasmid DNA, where the transcription initiates.

Chorn3 LNPs efficiently delivered siRNA into HeLa cells and led to the silencing of a disease-related gene (Plk1). Chorn3 Formulation (3), which gave highest cellular uptake of siRNA and highest GFP positive cells after the transfection of pcDNA-eGFP, induced most significant RNAi in HeLa cells, which is comparable to lipofectamine 2000. When the final concentration of siRNA was 5 nM, the mRNA level of Plk1 decreased 75%; at siRNA concentration 10 nM, Plk1 mRNA decreased 83%, and at siRNA concentration 25 nM, Plk1 mRNA wad reduced 90% (Figure 7). Chorn3 mediated siRNA delivery also induced DNA segregation and abnormal DNA condensation in the nucleus, typical phenotypes resulting from significantly decreased Plk1 protein level (Figure 8).

## 5. Conclusions

Using a straightforward synthesis approach, we successfully prepared peptidomimetic functionalized cationic cholesterol derivative Chorn3 with high yield. Formulation of Chorn3 with the helper lipid at a ratio of 5:1 (Chorn3: DOPC) containing 10% PEG2000-C-DMA gave the most potent LNP. Chorn3 LNP can efficiently deliver plasmid DNA to cancer cells and facilitate over 70% of the cell population to express GFP. Moreover, Chorn3 LNP can deliver siRNA to the cells with high efficiency, induces silencing of a disease-related endogenous gene at low siRNA concentrations. Collectively, our data suggest that the novel cholesterol-based cationic LNP may be a promising carrier for therapeutic nucleic acids including plasmid DNA and siRNA.

## Figures and Tables

**Table 1:** Calculation of volume (μL) of each Chorn3 stock solution used to form N/P (1:1).

Chorn3 Formulation	Stock Solution (μL) ^(a)^	RNase Free Water (μL) ^(b)^	Total Amine (N, nmol)	Stock siRNA (μL) ^(c)^	Total Phosphate (P, nmol)	RNase Free H_2_O (μL)	N/P
3:1:0.1	0.72	4.28	1.0	0.5	1.0	4.50	1.0
4:1:0.1	0.67	4.33	1.0				
5:1:0.1	0.64	4.36	1.0				
6:1:0.1	0.62	4.38	1.0				

^(a)^ Stock solution Chorn3 formulation (Chorn3 contains 4 free amino groups): 0.45 mg/mL. ^(b)^ Purchased from Takara Biotechnology Co., Ltd. Dalian, Liaoning, China. ^(c)^ Stock solution of siRNA (40 nucleotides double strand): 50 μM.

**Table 2:** Particle size ^(a)^ and zeta potential of Chorn3 lipid nanoparticles (LNP).

Chorn3 Formulation No.	Ratio of Components ^(b)^	Particle Size (d.nm)		Zeta Potential (mV)	
		−siRNA (PDI)	+siRNA (PDI)	−siRNA	+siRNA
(1)	3:1:0.1	110.4 (0.27)	178.4 (0.27)	43.9 ± 3.2	27.2 ± 2.5
(2)	4:1:0.1	87.6 (0.24)	167.4 (0.24)	44.5 ± 3.5	28.9 ± 1.8
(3)	5:1:0.1	91.4 (0.24)	151.8 (0.24)	46.9 ± 2.1	33.2 ± 2.1
(4)	6:1:0.1	123.7 (0.25)	197.8 (0.25)	41.2 ± 2.5	26.2 ± 3.1

^(a)^ Measured by dynamic light scattering; ^(b)^ Weight ratio of Chorn3 lipid, DOPC and PEG2000-C-DMA.

## Supplementary Materials

The following are available online. Figure S1: ^1^H NMR spectrum of cholesterol chloroformate and intermediate 1. Figure S2: Size distribution of Chorn3 LNPs before (A) and after (B) complexing with siRNA. Figure S3: Zeta potential of Chorn3 LNPs before and after complexing with siRNA.
