# Supplementary material for: Design of Peptidomimetic Functionalized Cholesterol Based Lipid Nanoparticles for Efficient Delivery of Therapeutic Nucleic Acids
Source: Molecules. 2019 Sep 19;24(18):3413. doi: 10.3390/molecules24183413 (PMC6767268; doi:10.3390/molecules24183413)
Supplement: Supplementary file 1 [file molecules-24-03413-s001.pdf]

Supplementary Materials

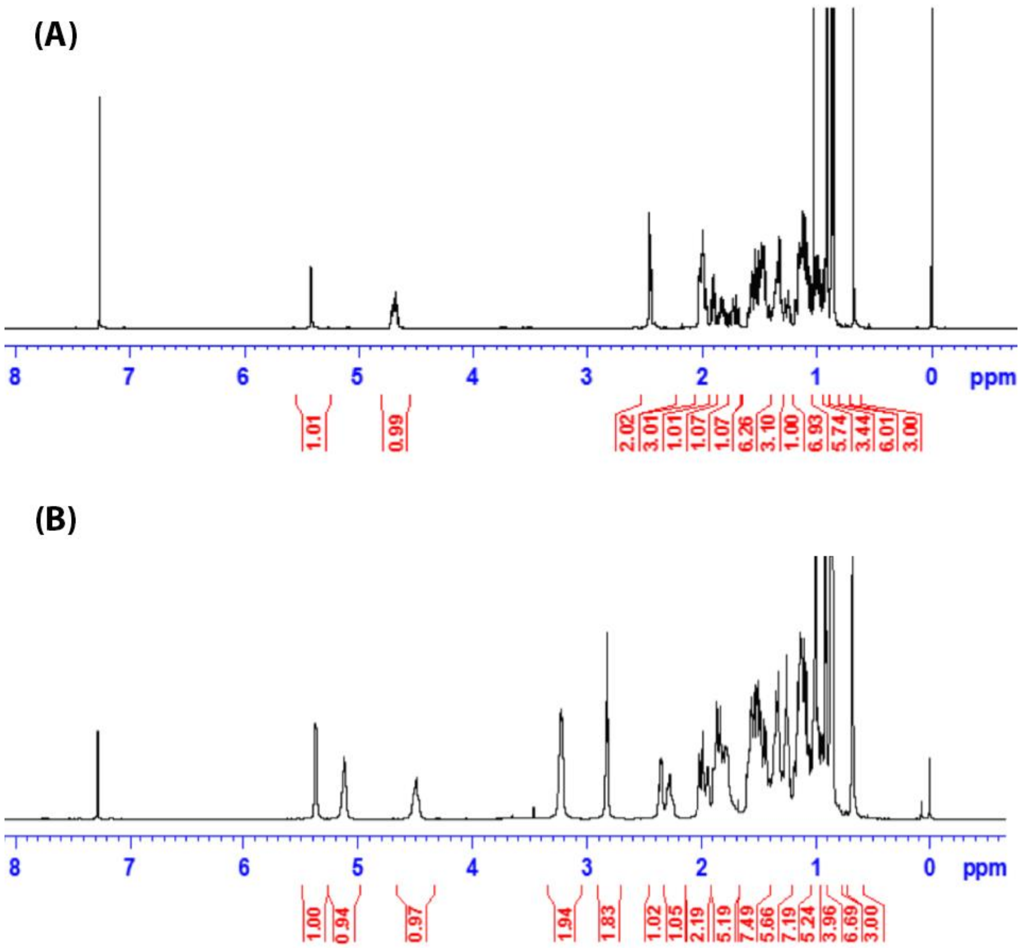

Figure S1. 1H NMR spectrum of cholesterol chloroformate (**A**) and intermediate 1 (**B**) in CDCl3.

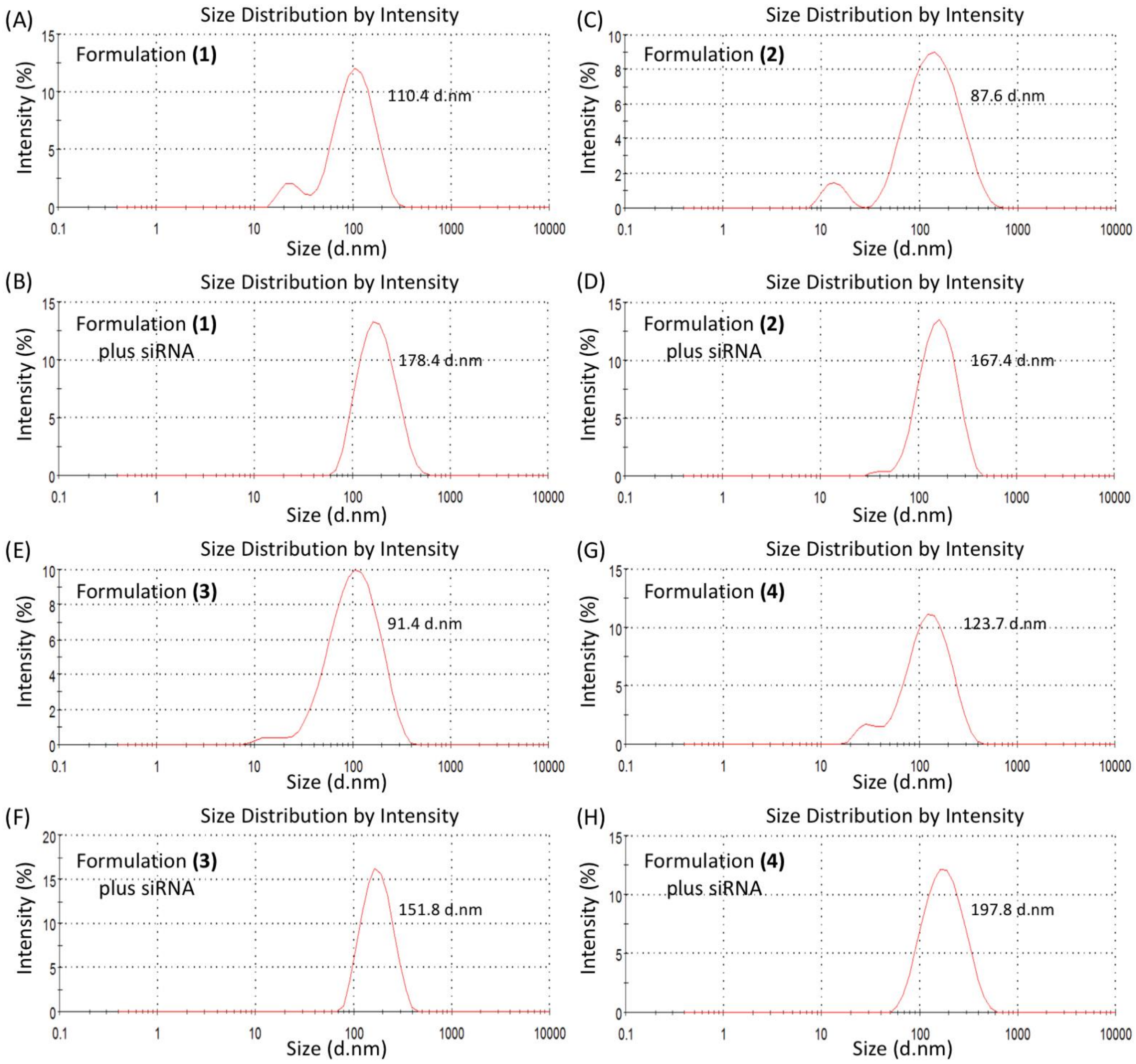

Figure S2. Size distribution of Chorn3 LNPs before and after complexing with siRNA.

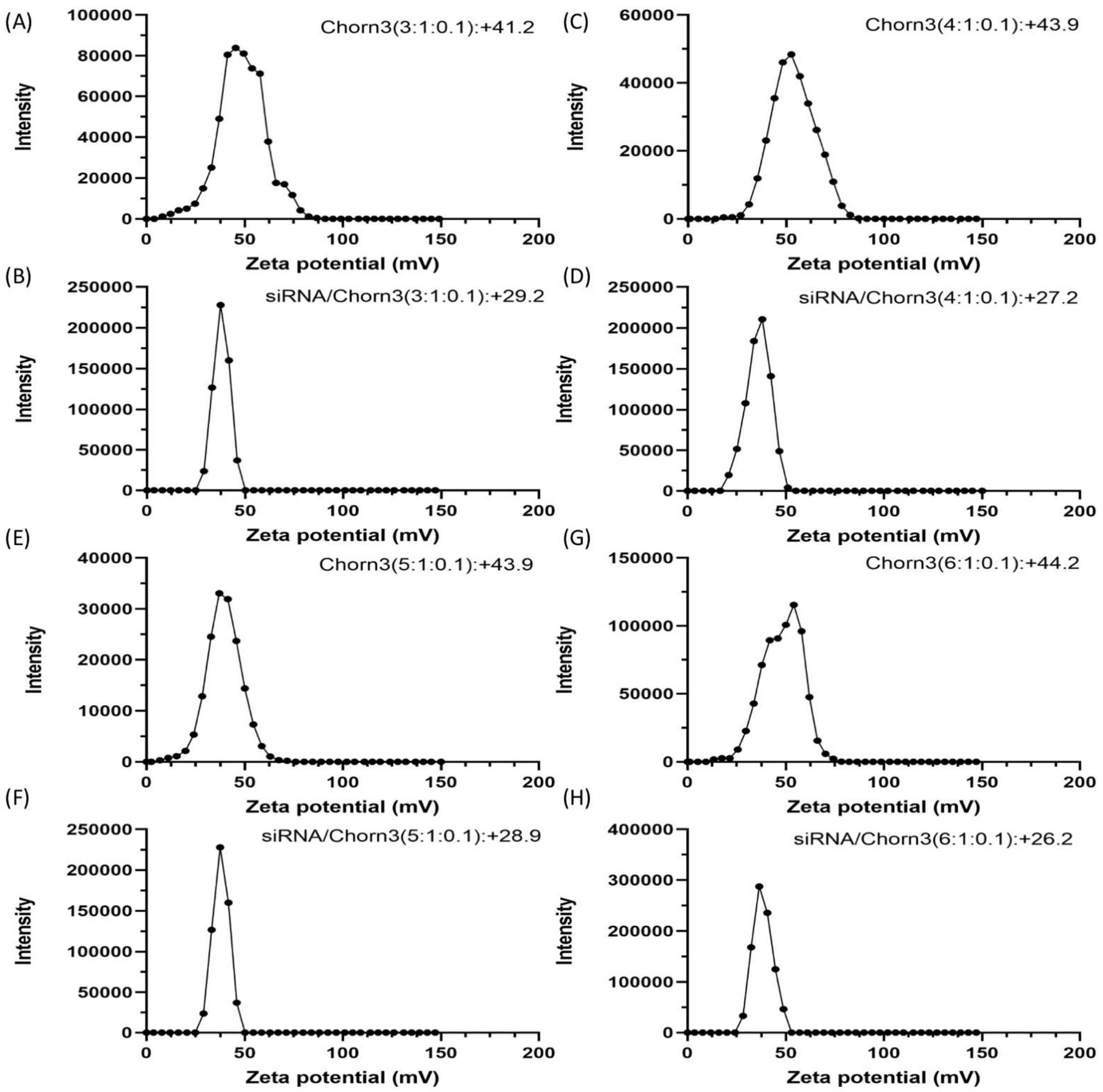

Figure S3. Zeta potential of Chorn3 LNPs before and after complexing with siRNA.
